# Biochar and compost amendments promote yield in specialty crops

**DOI:** 10.3389/fpls.2026.1849840

**Published:** 2026-06-19

**Authors:** Gayatri Mishra, David Roger Gang, Anna Berim, Douglas Collins, Bertram T. Jobson, Steven Seefeldt, Nathan Stacey, Neda Khosravi, Wendy Hoashi-Erhardt, Manuel Garcia-Perez

**Affiliations:** 1 Institute of Biological Chemistry, Washington State University, Pullman, WA, United States; 2 WSU Center for Sustaining Agriculture and Natural Resources, WSU Puyallup Research and Extension Center, Puyallup, WA, United States; 3 Laboratory for Atmospheric Research, WSU Department of Civil & Environmental Engineering, Pullman, WA, United States; 4 WSU Northwest Research and Extension Center, Mt. Vernon, WA, United States; 5 WSU Puyallup Research and Extension Center, Puyallup, WA, United States; 6 Biological Systems Engineering, WSU Biological Systems Engineering, Pullman, WA, United States

**Keywords:** basil, biochar, co-composting, metabolites, potato, regenerative, strawberry, sustainability

## Abstract

**Introduction:**

There is growing evidence for the positive impact of compost and biochar as soil amendments. However, the effect observed has varied widely, depending on the crop species and the particular biochar formulation. Few studies have evaluated more than one crop × biochar-based amendment combination.

**Methods:**

To further address this question, we conducted a series of experiments with several crop species grown in the greenhouse and/or in the field, comparing the impact on crop yield and quality of several biochar-based soil amendments, including raw biochar, co-composted biochar, and biochar admixed with compost, using more than one compost type.

**Results:**

The incorporation of compost, co-composted biochar, and a combination of compost and biochar led to an enhancement in the productivity of sweet basil and strawberries. The effects differed according to the type of compost, biochar, and crop. Two basil cultivars and three composts amended with biochar were used in a greenhouse study. Specific combinations of particularly co-composted biochar led to increased biomass output compared to controls. The same amendments to the soil did not markedly influence the phytochemical content of sweet basil cultivated in either field or greenhouse, suggesting that the quality of the final product was unaffected. Co-composted biochar led to biomass increases for both basil cultivars in a field-based experiment, but the increases were not identical, suggesting a genetic component to the plant’s response to the soil amendments. A strawberry experiment conducted in the greenhouse found that the average mass of single berries from plants grown with commercially produced compost plus 10% biochar was higher compared to several other treatments, particularly when it was co-composted. In a potato-based experiment, preliminary field trial data showed that co-compost amendments were the only amendment that increased potato crop production, but this was demonstrated only in fertilized split-plots.

**Discussion:**

Additional experiments will need to be conducted to evaluate the mechanisms responsible for the observed enhancement in yield for these various crop × biochar-based amendment combinations.

## 1 Introduction

The use of biochar-compost mixtures resulting from co-composting for agriculture is an emerging technology holding great promise (Agegnehu et al., 2017). A large multi-national study recently concluded that though the effects of biochar, compost, and biochar-compost blend (biochar added at the beginning or the end of the composting process) vary depending on feedstocks, the use of compost-biochar blends led to yields comparable to those obtained with mineral fertilizer, with a cumulatively lower environmental impact (Oldfield et al., 2018). Co-composted biochar increased yields, in comparison to compost-only, for oats (Schulz et al., 2013) and barley (Agegnehu et al., 2016). Clear benefits of co-composting in comparison to biochar added after composting (hereafter referred to as biochar plus compost) were observed in a study evaluating the biomass yield and nitrogen uptake of quinoa (Kammann et al., 2015). Our previous study indicated a greater biomass yield of sweet basil grown with co-composted biochar-compost mixtures as compared to treatments where biochar was added after composting (Gang et al., 2018). At the same time, some studies delivered the opposite results, suggesting that the effects of biochar-compost mixtures are highly dependent on specific application details (Gang, 2018). The benefits and drawbacks of biochar application may be related to its electrochemical properties (Chacon et al., 2017), its interactions with soil microbiota (Hol et al., 2017), and numerous other parameters (Agegnehu et al., 2017). Previous studies have also demonstrated that the effects of biochar on crop productivity, soil properties, and nutrient dynamics vary depending on feedstock type, soil conditions, crop species, and application strategy (Yamato et al., 2006; Van Zwieten et al., 2010; Bottoms et al., 2013; Agegnehu et al., 2015; Jay et al., 2015; Sun et al., 2019). For example, biochar can adsorb herbicides and reduce their efficacy. The bioavailability of the herbicides S-metolachlor and sulfentrazone was reduced more by high surface area biochar than by low surface area biochar at the same application rate (Graber et al., 2012). Surface adsorption and reduction in herbicide efficacy were lower in long-aged biochar (greater than 50 years) than in new biochar, possibly due to the formation of new surface functional groups (Cheng et al., 2014). Studies are being designed and executed to help clarify the impacts of different variables and enable the prediction of effects (Bonanomi et al., 2017). In addition, the properties of the agronomical environment play a crucial role in determining the effects of biochar and/or compost application. There is evidence that biochar amendment is most effective for degraded or nutrient-depleted soil (Agegnehu et al., 2017).

A study with strawberries grown in white peat with a 3% (by weight) biochar showed increased plant growth, reduced leaf and post-harvest fruit susceptibility to *Botrytis cinerea*, and a shift in the rhizosphere-associated microbial community (De Tender et al., 2016a, 2016). These effects were diminished when inorganic fertilizer and lime were co-administered, suggesting higher biochar efficacy under limited nutrient availability. In the same study, biochar did not affect lettuce grown in sandy loam soil (De Tender et al., 2016b). A time-course experiment determined that the effects of biochar on the microbial community were evident from week six of growth onwards (De Tender et al., 2016a). Notably, Harel et al. (2012) demonstrated biochar-related enhancement of strawberry plant resistance to *Botrytis* infection in an entirely different experimental setting. It has been documented for both biochar and compost that feedstock has an important effect on the quality of the resulting product (Jindo et al., 2014; Agegnehu et al., 2017).

In this study, we evaluated three types of biochar-compost products resulting from combinations of two types of compost and two sources of biochar. The goal of the experiment was to compare the effects of different biochar-compost products on crop yield and quality. Three crops were used in this current study. Sweet basil is an important high-value crop in Washington State (Gang et al., 2018). In addition to biomass productivity and aroma composition, an important quality parameter for a culinary herb like sweet basil, we assessed the effect of biochar-compost amendments on the accumulation of antioxidant polyphenolics that add nutritional value for the consumer. The second specialty crop in this study was strawberry (*Fragaria x ananassa*). The Pacific Northwest region, including Oregon, Washington, and British Columbia (Canada) produces approximately 32 million pounds annually of strawberries, which are mostly processed or sold via direct market (Samtani et al., 2019). The predominant areas of commercial strawberry production are in the Skagit and Nooksack valleys in Washington. Albion is the major day-neutral cultivar grown. The third crop examined in this study was potato, which is a major crop grown in Washington, with 169,000 acres of potatoes harvested in the state in 2016. The Skagit Valley of Washington is a major producer of potatoes for the fresh market. The study evaluated the impact of biochar, compost, co-composted biochar, and biochar plus compost on the productivity and quality of sweet basil in the greenhouse and the field, the productivity of strawberries in the greenhouse and the field, and the productivity of potatoes in the field. The study assessed biomass productivity, aroma composition, an important quality parameter for a culinary herb like sweet basil, and the effect of biochar-compost amendments on the accumulation of antioxidant polyphenolics that add nutritional value for the consumer.

## 2 Materials and methods

### 2.1 Compost generation

#### 2.1.1 Composts produced at the WSU composting facility in 2017

The generation of these composts was previously described by Gang et al. (2018). The biochar used was obtained from Amaron Energy (Salt Lake City, UT), and produced using a patented rotary kiln unit. This compost was referred to as “Washington State University (WSU) 2017 compost.

#### 2.1.2 Composts produced at the WSU composting facility in 2018

In 2018, biochar (Oregon Biochar Solutions, White City) was used to produce compost at the WSU composting facility. Biochar for the project was generated in two modified biomass boilers that convert Douglas fir (*Pseudotsuga menziesii*) and pine (*Pinus* spp.) forestry residuals into biochar at 871^○^C in a low oxygen environment (Oregon Biochar Solutions, White City, OR). The physicochemical properties of the biochar were characterized prior to use, including elemental composition, proximate analysis, and surface area measurements. Carbon content ranged from 85.5–88.2%, nitrogen content ranged from 0.76–0.81%, and fixed carbon content ranged from 84.7–88.0%. Surface area values determined using N2 adsorption ranged from 521–547 m2 g−1. Overall, twelve compost piles were made. Each compost pile contained: 4.5 yards of screened manure solids from the WSU dairy, 4.5 yards of dairy bedding straw and manure, 0.5 yards of ground clean green (woody) yard trimmings, and 0.5 yards of food waste from the WSU dining commons. These feedstocks were selected to provide balanced carbon and nitrogen inputs during composting. These components were mixed and stacked in separate 10-yard piles on June 4 and 5. At least one pile from each treatment was constructed on a different day to minimize potential day effects associated with environmental variation during pile establishment. At the initiation of the composting process, biochar was mixed at either 2.5%, 5% or 10% (by volume). Hereafter, percentages referred to in biochar mixtures indicate percent by volume. Each treatment level was assigned to three piles; the remaining three piles contained no biochar (compost control). Thus, each treatment consisted of three biological replicate compost piles. The compost piles were monitored for temperature (maintained >57 °C) and bulk density throughout the composting process. Maintaining thermophilic temperatures ensured active composting and pathogen reduction. By the end of the composting period, the piles had reduced to about 5 yards each. This compost was referred to as “WSU 2018 compost”.

#### 2.1.3 Composts produced at Lenz Enterprises in 2018

Lenz Enterprises is a commercial compost operation that employs a three-stage composting process that uses municipal yard and food waste in an aerated static pile. Piles were turned once during the aeration period, then moved out of the aerated composting bay and allowed to compost further and cure. The biochar co-composted amendment was made simultaneously with the same feedstocks, with the addition of 5% biochar by volume (Oregon Biochar Solutions). Both piles were composted in the same 800 yd^3^ aerated bin and were marked to keep them separated, with the biochar co-compost having a starting volume of 200 yd^3^. This compost was referred to as “Lenz compost”. Compost air emissions were sampled on days 3, 7, 10, 16, and 22 (WSU) and days 3, 7, 11, and 31 (Lenz). Sample collection and analyses were carried out as described previously by Gang et al. (2018).

#### 2.1.4 Biochar and compost-generated properties

The elemental and proximate content of three lots of the OBS biochar were determined (**Tables 1**, **2**). Elemental analysis was performed using a TRUSPEC-CHN^®^ (LECO, US) elementalanalyzer. Briefly, 0.05 g oven dried samples were used to determine total C, N and hydrogen (H). Oxygen (O) mass fraction was determined by subtracting the ash, C, N, and H mass fraction from the total mass of the sample. Fixed carbon, volatiles, and ash mass fraction were determined by using a high-temperature muffle furnace, Isotemp^®^, and a thermo-gravimetric analyzer (TGA), SDTA851e (Mettler Toledo, US). Briefly, 1.5 g of oven-dried samples were weighed into a pre-weighed crucible and heated in air at 848 K for 12 h to determine the ash fraction. Volatiles and fixed carbon were determined by thermogravimetric analysis under nitrogen. Additionally, some of the properties of the same biochar and simultaneously of the Lenz compost and co-compost were measured by the Collins team (**Supplementary Table 1**).

**Table 1:** Elemental analysis of OBS (Oregon biochar solutions) biochar.

Sample	Carbon%	Hydrogen %	Nitrogen %	Sulfur %	Oxygen %*
Lot_01	85.471	0.861	0.782	0.033	6.040
Lot_02	88.216	0.776	0.759	0.025	4.568
Lot_03	88.136	0.754	0.809	0.037	3.281

**Table 2:** Proximate analysis of OBS biochar and surface area and pore volume of OBS biochar.

	Proximate analysis			
Sample	Volatile Carbon %	Fixed Carbon %	Ash %	Moisture %
Lot_01	10.481	84.684	4.835	2.226
Lot_02	9.270	88.025	2.706	3.320
Lot_03	9.911	86.431	3.658	3.739

	Surface area and pore volume				
Sample	Surface area obtained by using CO_2_ gas (SaCO_2_) m^2^/g	Surface area obtained by using N_2_ gas (SaN_2_) m^2^/g	Micropore volume (V_micro_ cm^3^/g)	Mesopore volume (V_meso_ cm^3^/g)	Total volume (V_Total_cm^3^/g)
Lot_01	549.3	520.9	0.22	0.08	0.30
Lot_02	580.3	547.2	0.23	0.06	0.29
Lot_03	592.9	531.5	0.24	0.06	0.30

Selected physicochemical properties of the biochar, compost, and co-compost included carbon content, nitrogen content, moisture, ash content, and C:N ratio. The OBS biochar contained approximately 87 wt. % carbon and exhibited a high carbon-to-nitrogen (C:N) ratio (~112), consistent with carbon-rich pyrolyzed woody biomass materials. In contrast, compost and co-compost materials had substantially lower C:N ratios (~15), reflecting greater nitrogen availability and more advanced organic matter decomposition in the composted amendments. Moisture content of compost and co-compost exceeded 50%, while ash content ranged between 38–39%. These physicochemical differences among amendments were expected to influence nutrient retention, microbial activity, and soil physicochemical behavior following soil incorporation.

### 2.2 Greenhouse and field trials

The sources of compost, co-compost, and biochar for each of the experiments included in thisstudy are shown in **Supplementary Table 2**.

#### 2.2.1 Basil greenhouse trial

The greenhouse basil experiment used the same soil mix recipe as in the earlier study (Gang et al., 2018), which consisted of 37.5% potting soil(Sunshine #4, Sun Gro Horticulture, Agawam, MA), 12.5% perlite, 25% field soil (collected from a field at HerbCo International’s main site in Duvall, WA), and 25% compost (with or without biochar) (all percentages by volume). The inclusion of agricultural field soil in the greenhouse substrate was intended to provide a more representative physicochemical and microbial environment relative to sterile potting substrates alone. Pots for different treatments were placed randomly within the greenhouse area and rotated regularly throughout the experiment to minimize positional and edge effects. For the basil greenhouse experiment, the same two sweet basil cultivars (Eleanora and Thai Siam Queen, abbreviated TSQ) were grown for the first study (Gang et al., 2018). The experiment took place between 28 November 2018 (seeds planted) and 4 February 2019 (harvest). Ten plants were grown for each of the 13 treatments, with three different sources of composts described above. The treatments are summarized in **Supplementary Table 3**. As in the earlier study (Gang et al., 2018), the addition of biochar after composting was based on the initial volume of compost to mimic the proportions during co-composting. Plants were grown as described earlier (Gang et al., 2018) except for the early stage which occurred as follows. The seeds were germinated in peat pellets (4 seedlings/pellet) and thinned after germination to 1 seedling/pellet before being transplanted into soil. Until the transplant they stayed in a growth chamber kept at 27/22 °C day/night, and 12/12 hour light cycle, and 80% relative humidity for 26 days. After transplanting to larger pots, the plants were placed in the greenhouse and grown with supplemental lighting and under the conditions previously described for an additional 40 days of growth, at which time they were photographed and harvested 66 days after planting the seeds. At harvest, each plant was cut off at base (about 1.5–2 cm from soil), fresh weight recorded, and leaf material collected and stored as described earlier (Gang et al., 2018).

#### 2.2.2 Basil field trial

Sweet basil (*Ocimum basilicum* L. ‘Genovese’) was grown in the field. The plants were grown at an organic farm, Footehills Farm in Colbert, Washington, which has been growing crops on site for many years with prior application annually of compost produced on-site. The plots were located in the middle of the field, surrounded by other plants to avoid edge effects. Treatment plots were distributed throughout the production area to reduce local positional effects, and all plants were maintained under the same irrigation and crop management conditions used by the grower. As each basil seedling was transferred to the field, a hole of approximately 3 liters (~20 cm deep) was dug by hand with a small trowel, with approximately 500 ml of compost or co-composted biochar (of one of the various treatments) mixed in to the loose soil in the hole, and the seedling then placed in the hole. Total percent of compost added to each hole was, therefore, approximately 17% of the total volume. Overall, control plants grown in soil without any amendment were compared to six treatments: plants grown with WSU 2017 compost without biochar, Footehills compost without biochar, WSU 2017 compost co-composted with 2.5% biochar (total in co-compost mix by volume), WSU 2017 compost co-composted with 5% biochar (total in co-compost mix by volume), WSU 2017 compost mixed plus 2.5% biochar (total in co-compost mix by volume), or WSU 2017 compost mixed with 5% biochar (total in co-compost mix by volume). Plants were harvested on 17 August 2018, about three months after planting, when they reached the typical size harvested by the grower (Thom Foote from Footehills Farm); this was typically just as inflorescences started to develop. Each plant was cut off at the base, weighed immediately due to concerns about mass loss from wilting after harvest, photographed, and the two youngest, fully mature leaf pairs were collected and frozen on dry ice for chemical analysis.

#### 2.2.3 Basil metabolite analysis

Extraction and analysis of non-volatile phytochemicals from sweet basil leaves (with a focus on major phenolic antioxidants) by liquid chromatography-mass spectrometry (LC-MS) were carried out as described previously (Gang et al., 2018), with the modification that methanol was used as an extraction solvent instead of 85% aqueous ethanol. In addition, ultrasonication time was shortened from 30 to 15 minutes for the greenhouse-grown Eleanora basil material. Those extracts were incubated on an orbital shaker for 60 minutes at room temperature after the ultrasonication. Extraction and analysis of sweet basil volatile aroma compounds followed the procedure described previously (Gang et al., 2018).

#### 2.2.4 Strawberry greenhouse trial

Bare-root strawberry (*Fragaria* x *ananassa*) plants of the day-neutral cultivar ‘Albion’ were purchased from Lassen Canyon nursery (Redding, CA). The plants were potted on 27 June 2018. All fruit, flowers, and runners were removed at transplant and for two weeks thereafter to promote root and leaf growth. This early removal of reproductive tissues was performed to standardize vegetative establishment prior to fruit production. The plants were grown in the greenhouse under ambient lighting, which was supplemented by high-sodium pressure lights that activated when the light intensity dropped below 200 µmol m^-2^·s^-1^. The day temperature was maintained at 21-24 °C, and the night temperature at 17-20 °C. Treatments were arranged in a randomized greenhouse layout and pots were repositioned regularly during cultivation to minimize positional effects associated with greenhouse environmental variability. One-half of the plants received general-purpose 20-10–20 Peters Professional Liquid Feed fertilizer with chelated iron (Sprint 330), Epsom salts, and micronutrients 2–3 days per week at 200 ppm. There was a total of 8 treatments, with 10 plants per treatment. Five treatments were based on WSU 2017 compost: 1) compost without biochar amendment, 2) co-composted with 2.5% Amaron Energy biochar, 3) co-composted with 5% Amaron Energy biochar, 4) compost plus 2.5% Amaron Energy biochar, and 5) compost plus 5% Amaron Energy biochar. The other 3 treatments were based on Lenz compost: 6) compost without biochar, 7) co-composted with 2.5% Oregon Biochar Solutions char, or 8) compost plus 5% Oregon Biochar Solutions biochar (mismatched biochar concentrations between treatments 7 and 8 are due to a miscommunication). Fruit was collected from each plant as it ripened; in total, there were 22 harvests between 4 August 2018 and 31 October 2018. At each harvest, the total weight of fruit and the number of berries were recorded.

#### 2.2.5 Strawberry field trials

Research plots were installed at Washington State University’s Puyallup Research and Extension Center and planted with strawberry (*Fragaria* x *ananassa* Duch.) cultivar ‘Albion’, a common commercial cultivar in the Pacific Northwest. Strawberry growers amend their soils with supplementary nitrogen in the form of fertilizer. Therefore, we included an additional component in the experimental design (i.e., fertilizer), allowing us to provide a comprehensive analysis of two management strategies on which realistic expectations and grower recommendations can be based. Including an unamended control, five treatments: biochar alone, compost alone, co-compost, and a field-mixed biochar plus compost blend, were surface amended in a randomized split-plot design, which was replicated four times at each location. The randomized split-plot design enabled evaluation of both amendment effects and amendment × fertilizer interactions under field production conditions representative of commercial strawberry cultivation systems in Washington State. At both sites, the main plot factor was nitrogen fertilizer (i.e., fertilized or unfertilized), and the sub-plot factor was amendment (e.g., biochar). Amendments were applied to meet a target rate of 8.75 Mg ha^-1^ organic carbon (C) and are listed in **Table 3**. Following the amendment, soils were tilled to a depth of 15 cm and then prepared for planting. This incorporation depth was selected to ensure relatively uniform amendment distribution within the primary root zone. Raised bed research plots were covered with black polyethylene and greenhouse grown bare root plugs were planted, by hand, on 10 July 2018 in four rows per 11.1 m^2^ plot sub-plot and immediately irrigated. Calcium nitrate (15.5-0-0) was applied by fertigation to fertilized plots in weekly applications of 2 kg N ha^-1^, totaling 20 kg N ha^-1^. All plots received an application of Malathion at 295.7 g ai ha^-1^. Irrigation was applied as necessary to research plots utilizing drip irrigation. Strawberry fruit was harvested and weighed weekly beginning 10 August 2018 and continuing through 3 October 2018. Yield data from each of the 9 collections was combined and analyzed as cumulative yield.

**Table 3:** Application rates for each of the four soil amendments applied to research plots in Puyallup and Mount Vernon, Washington.

Amendment	Dry Mg ha^-1^	C Mg ha^-1^	m^3^ ha^-1^	C:N ratio
Biochar (OBS)	8.8	7.65	40.2	112
Compost (Lenz)	27.3	8.75	54.9	15
Co-compost	27.4	8.75	54.9	15
Biochar + compost	18.7 (4.4 + 13.7)	8.21	47.6	26

#### 2.2.6 Potato field trial

Research plots were installed at the Mount Vernon Northwest Washington Research and ExtensionCenter and planted with potato (*Solanum turberosum* L.) cultivar‘Chieftain’. The experimental design and treatments were as described for the strawberry field trials above. The experiment employed a randomized split-plot design with four replicate blocks. As with the strawberry field trial, fertilizer was included as an additional component of the potato field trial. This design enabled evaluation of amendment performance under contrasting nutrient availability conditions relevant to commercial potato production systems. Following amendment, soils were tilled to a depth of 15 cm and then prepared for planting. Using a two-row planter, potatoes were planted to a depth of 15 cm on 7 June 2018 and hilled 2 July 2018 in four rows per 23.6 m^2^ sub-plot. All plots received applications of Outlook (dimethenamid) at 941 g ai ha^-1^ and Tricor 75 DF (metribuzin) at 561 g ai ha^-1^ and supplementary macro- and micronutrient additions: muriate of potash (0-0-60) at 111 kg·ha^-1^, boron 15% granular at 5.6 kg·ha^-1^, and procote BMZ at 111.2 kg·ha^-1^. Unfertilized plots received 0-45–0 at 77.34 kg·ha^-1^ and fertilized plots received the nutrients at rates listed in **Supplementary Table 4**, which totaled 108.6 kg N·ha^-1^. Irrigation was applied as necessary to research plots utilizing overhead irrigation. Irrigation was managed to maintain agronomically relevant soil moisture conditions throughout crop establishment and tuber development. Weed cover was measured on 30 July 2018. Potatoes were harvested on 10 September 2018, and 3-plant subsamples (2 plot^-1^), including above and below ground biomass, were collected, separated by tuber and leaf tissue, and later weighed. Reported results are the average tuber weight per sub-plot.

### 2.3 Statistical analysis

All statistical analyses were conducted using biological replicate measurements for each treatment. Depending on the experimental design, data were analyzed using one-way or two-way analysis of variance (ANOVA). For experiments involving multiple factors, including cultivar and amendment interactions, factorial ANOVA models were applied. When ANOVA indicated significant treatment effects, means were separated using Tukey’s honestly significant difference (HSD) *post hoc* test at p < 0.05. Data are presented as mean ± standard error of the mean (SEM), unless otherwise stated. Pots and treatments in greenhouse experiments were randomized and rotated regularly to minimize positional effects. Where appropriate, treatment comparisons were performed separately for individual crop systems and growing conditions. Statistical analyses and graphical visualizations were conducted using standard statistical software packages, including Microsoft Excel and R.

## 3 Results

### 3.1 Impact of biochar amendment on basil productivity and quality

#### 3.1.1 Basil greenhouse trial

**Figure 1:**
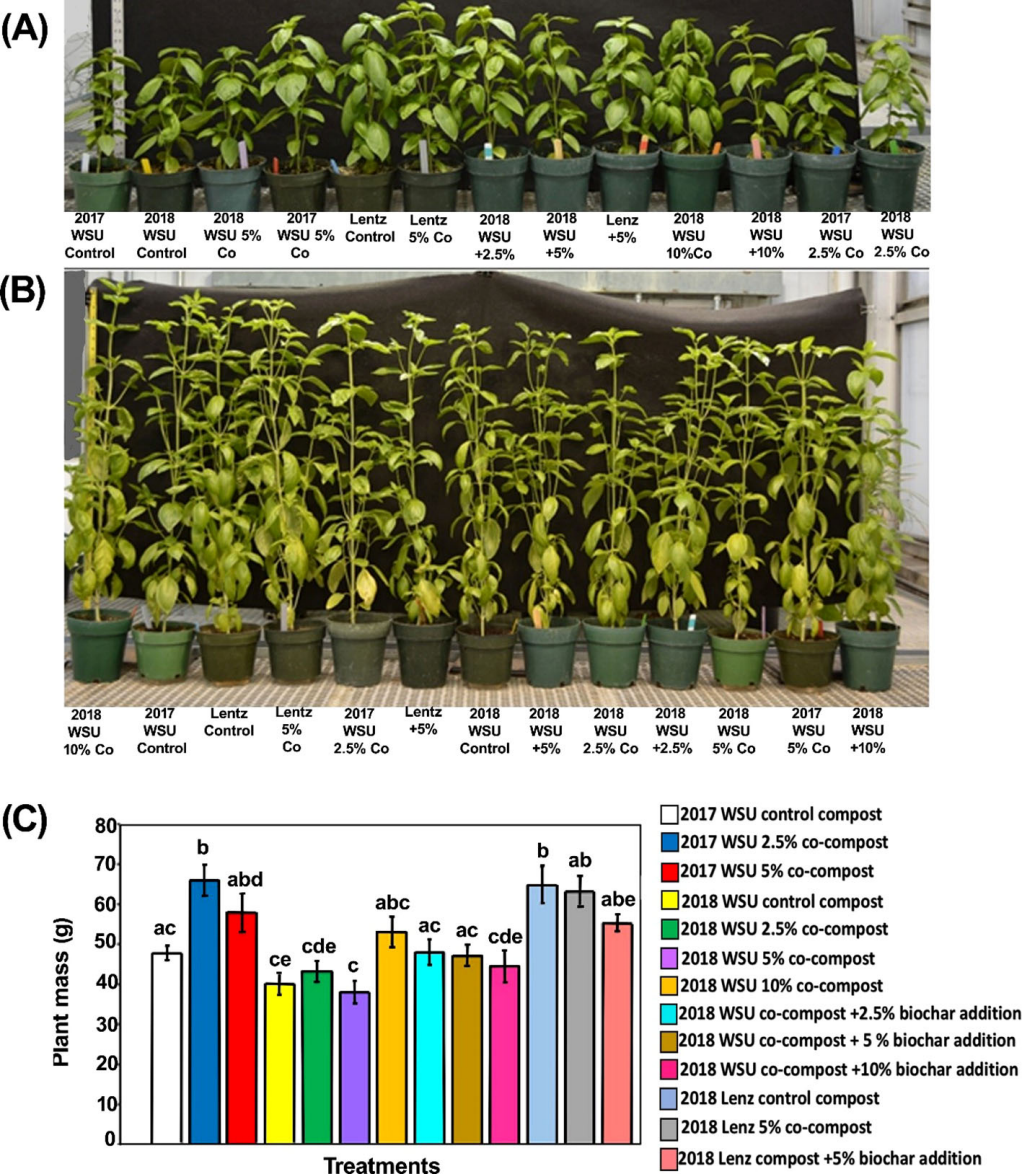
The effect of biochar amendments on biomass production in greenhouse-grown Eleanora sweet basil. Tallest plants from each treatment were photographed on **(A)** 9 January 2019 and **(B)** 4 February 2019. On 4 February, plants were lined up in the order of decreasing height **(C)**. Biomass of all plants for each treatment is shown as mean ± SEM (n=10). Different letters indicate statistically significant differences according to Tukey’s HSD test (p < 0.05). In **(A)** and **(B)**, “+5%” indicates 5% biochar added after composting and “5% co” indicates 5% biochar co-composted (added before composting).

**Figure 2:**
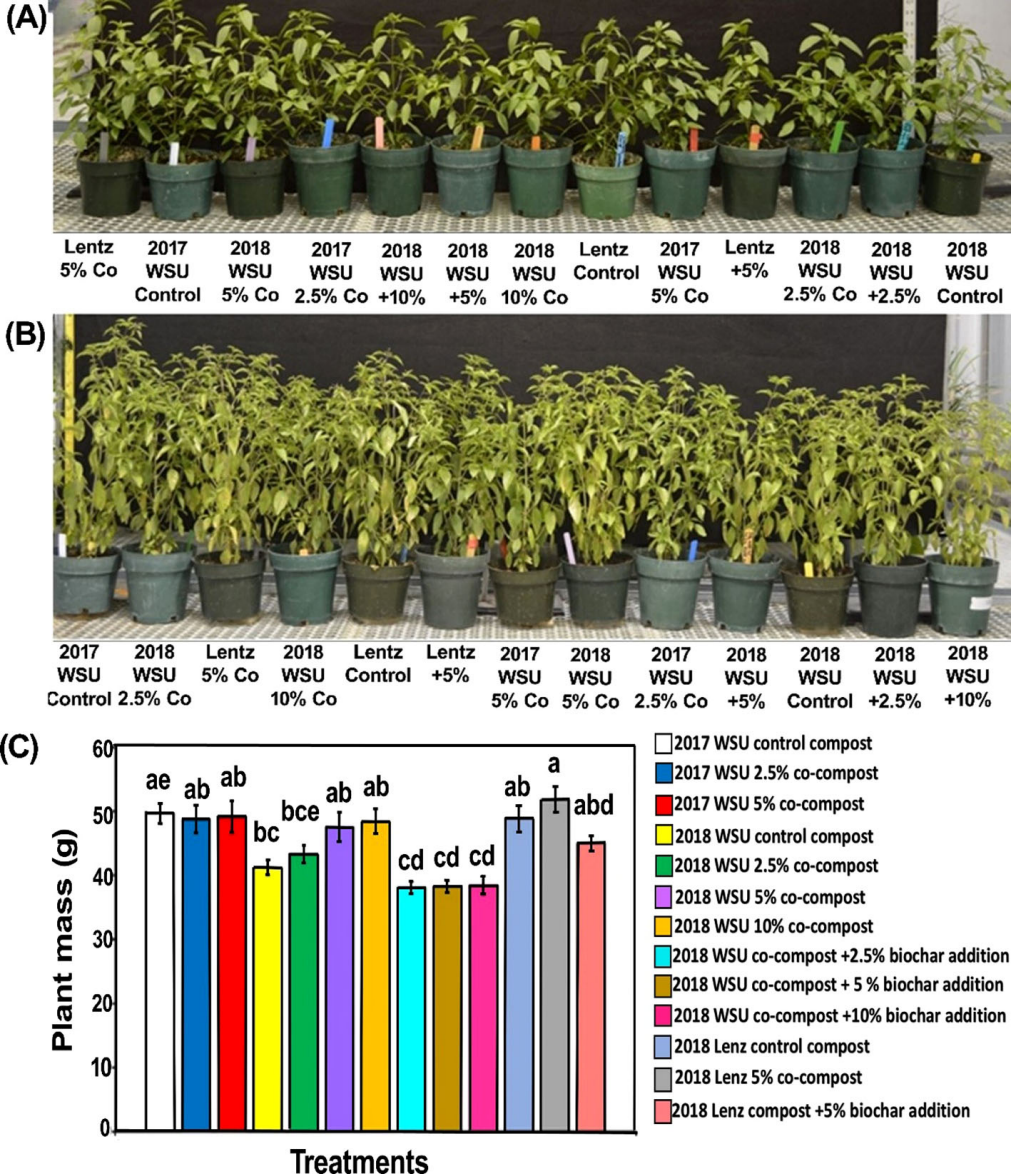
The effect of biochar amendments on biomass production in greenhouse-grown Thai Siam Queen sweet basil. The tallest plants from each treatment were lined up in the order of decreasing height and photographed on **(A)** January 9, 2019, and **(B)** February 4, 2019. **(C)** Biomass of all plants for each treatment is shown as mean ± ± SEM (n=10). Different letters indicate statistically significant differences according to Tukey’s HSD test (p < 0.05). In **(A)** and **(B)**, “+5%” indicates 5% biochar added after composting, and “5% co” indicates 5% biochar co-composted (added before composting).

The experiment with greenhouse-grown basil aimed to reproduce previous findings and evaluate the effects of two other compost types produced at different facilities. Basil plants of the Eleanora and Thai Siam Queen (TSQ) varieties were grown in the greenhouse under the same conditions as in March–May 2017, but at a different time of the year (late November to early February). Thirteen treatments with three different types of compost were produced at WSU in 2017 and 2018 and at Lenz in 2018. Composts produced in 2018 were co-composted with the same biochar from Oregon Biochar Solutions. The plants of both varieties grew faster than in the earlier trial, reaching harvest-suitable size about 40 days after transplanting (**Figures 1A**, **2A**). To monitor further growth, they were left to grow until 66 days after transplanting, when they were harvested. At that time, Eleanora basil plants still appeared healthy (**Figure 1B**), except for the first true leaf pair, which was often yellowed and sometimes wilted, while TSQ plants showed clear signs of leaf senescence (**Figure 2B**).

**Figure 3:**
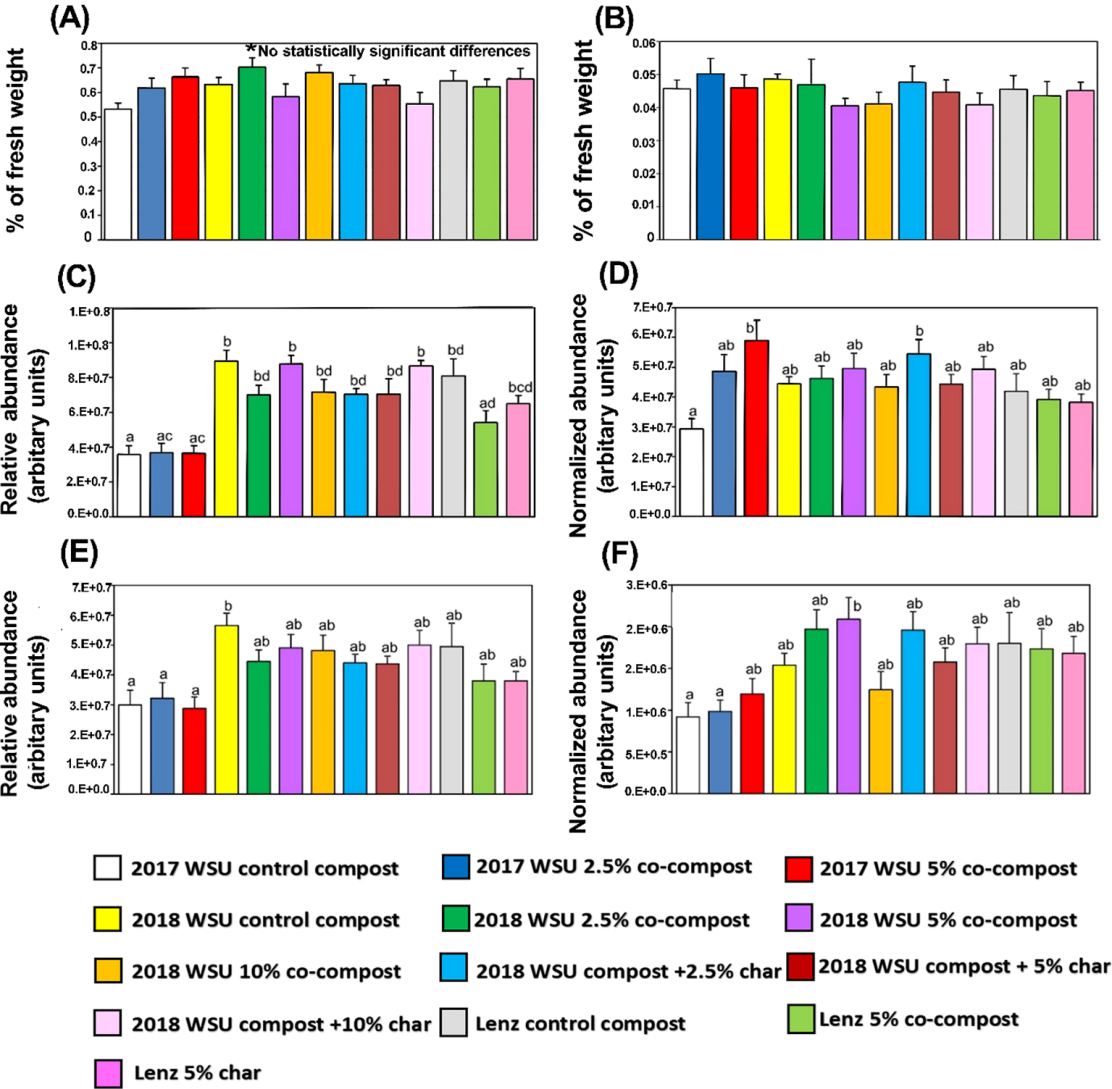
The effect of biochar amendments on antioxidant phenolics and volatile aroma component accumulation in Eleanora sweet basil. Contents of **(A)** rosmarinic acid and **(B)** chicoric acid in the two youngest mature leaves are presented as % of fresh weight. Contents of **(C)** eugenol, **(D)** eucalyptol, **(E)** linalool, and **(F)** ocimene in the two youngest mature leaves are presented as relative abundance (arbitrary units), normalized to internal standard and fresh tissue weight. Note that “+5% char” indicates 5% biochar added after composting and “5% co-compost” indicates 5% biochar co-composted (added before composting). Shown data are means ± SEM. (n=10). Different letters indicate statistically significant differences according to Tukey’s HSD test (p < 0.05). Statistical differences were evaluated using one-way ANOVA (Tukey’s *post-hoc* HSD test, p<0.05).

The amendments resulted in biomass yield differences across treatments and cultivars. Application of Lenz compost, alone, co-composted with biochar, or mixed with biochar post-composting, did not affect biomass yield in either Eleanora or TSQ (**Figures 1C**, **2C**). WSU 2017 compost, alone or co-composted with 2.5% or 5% Amaron Energy biochar, did not affect the biomass yield of TSQ (**Figure 2C**). In Eleanora, the average mass of plants grown with WSU 2017 compost co-composted with 2.5% biochar was greater compared to plants grown with WSU 2017 compost alone, while co-compost with 5% biochar resulted in a further biomass yield increase (**Figure 1C**). The vegetative yield of Eleanora basil with WSU 2018 compost, alone, co-composted with biochar, or admixed with biochar at different amounts, was similar. In TSQ, biomass yield from plants grown with WSU 2018 compost admixed with all three levels of biochar was lower than yields with WSU 2018 compost co-composted with 5% or 10% biochar (**Figure 2C**). The accumulation of antioxidants was evaluated in Eleanora plants. The overall abundances of the two major phenolics, rosmarinic acid and chicoric acid, were similar across treatments (**Figures 3A**, **B**). One-way ANOVA (p = 0.05) showed no significant differences between treatments, although rosmarinic acid appeared higher in plants grown with 5% co-composted biochar compared to the WSU 2017 control (**Figure 3A**). The addition of Lenz compost, with or without biochar, also resulted in slightly higher biomass, but differences were not statistically significant. To evaluate the effect of amendments on basil quality, volatile aroma compounds were analyzed in Eleanora plants. The major components detected by GC–MS were eucalyptol, linalool, eugenol, and ocimene. All plants exhibited similar overall profiles (**Figures 3C–F**). Eugenol and linalool were less abundant in plants treated with amendments containing WSU 2017 compost (**Figures 3C**, **E**). When considered separately by compost type, no consistent treatment effects were observed. Compared to WSU 2017 compost, eugenol accumulation was higher in plants grown with WSU 2018 compost, WSU 2018 co-composted with 5% OBS biochar, and WSU 2018 + 10% OBS biochar (**Figure 3C**). Linalool was more abundant in plants grown with WSU 2018 compost compared to all WSU 2017 treatments (**Figure 3E**). Eucalyptol was more abundant in plants grown with WSU 2017 compost co-composted with 5% Amaron Energy biochar compared to WSU 2017 compost without biochar (**Figure 3D**). Ocimene was lower in WSU 2017 compost and WSU 2017 compost co-composted with 2.5% Amaron Energy biochar compared to WSU 2018 compost co-composted with 5% OBS biochar (**Figure 3F**).

#### 3.1.2 Basil field trial

**Figure 4:**
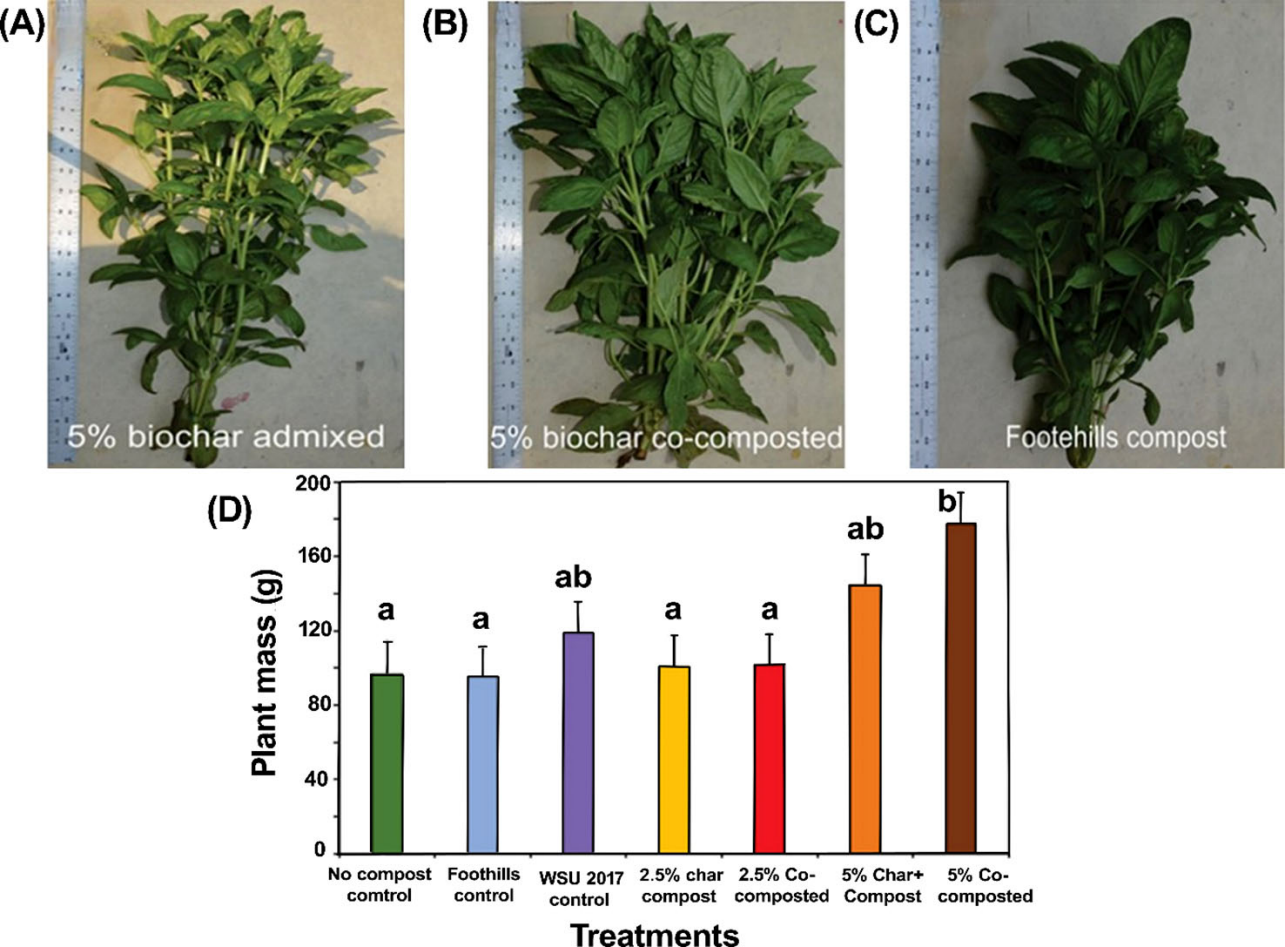
Organic Genovese basil grown at Footehills Farm (Colbert, Washington) with addition of: **(A)** WSU 2017 compost, mixed with 5% Amaron Energy biochar post-composting; **(B)** WSU 2017 compost, co-composted with 5% Amaron Energy biochar; or **(C)** Footehills organic compost. **(D)** Analysis of quantitative differences between treatments revealed that 5% biochar, either added post-composting or co-composted, led to significant increases in biomass compared to controls, with the latter leading to an almost doubling (80% greater) of the biomass. Values represent mean ± SEM (n = 10). Different letters indicate statistically significant differences according to one-way ANOVA followed by Tukey’s HSD test (p < 0.05).

The treatments did not reveal injury differences among plants, i.e., none caused chlorosis or leaf malformation, and all plants appeared healthy (**Figures 4A–C**). Both biomass production (yield) and chemical composition (herb quality) were determined. The fresh plant mass yield was very similar for four treatments: plants grown without or with Footehills compost and plants grown with WSU 2017 compost amended with 2.5% biochar. The addition of WSU 2017 compost amended with 5% biochar before composting led to higher plant mass than these four treatments (ANOVA with Tukey *post-hoc* HSD test, p < 0.05). Average biomass yield was also higher when 5% non-composted biochar with WSU 2017 compost, or WSU 2017 compost by itself, was added to the soil, but not different in comparison to all other treatments (**Figure 4D**).

**Figure 5:**
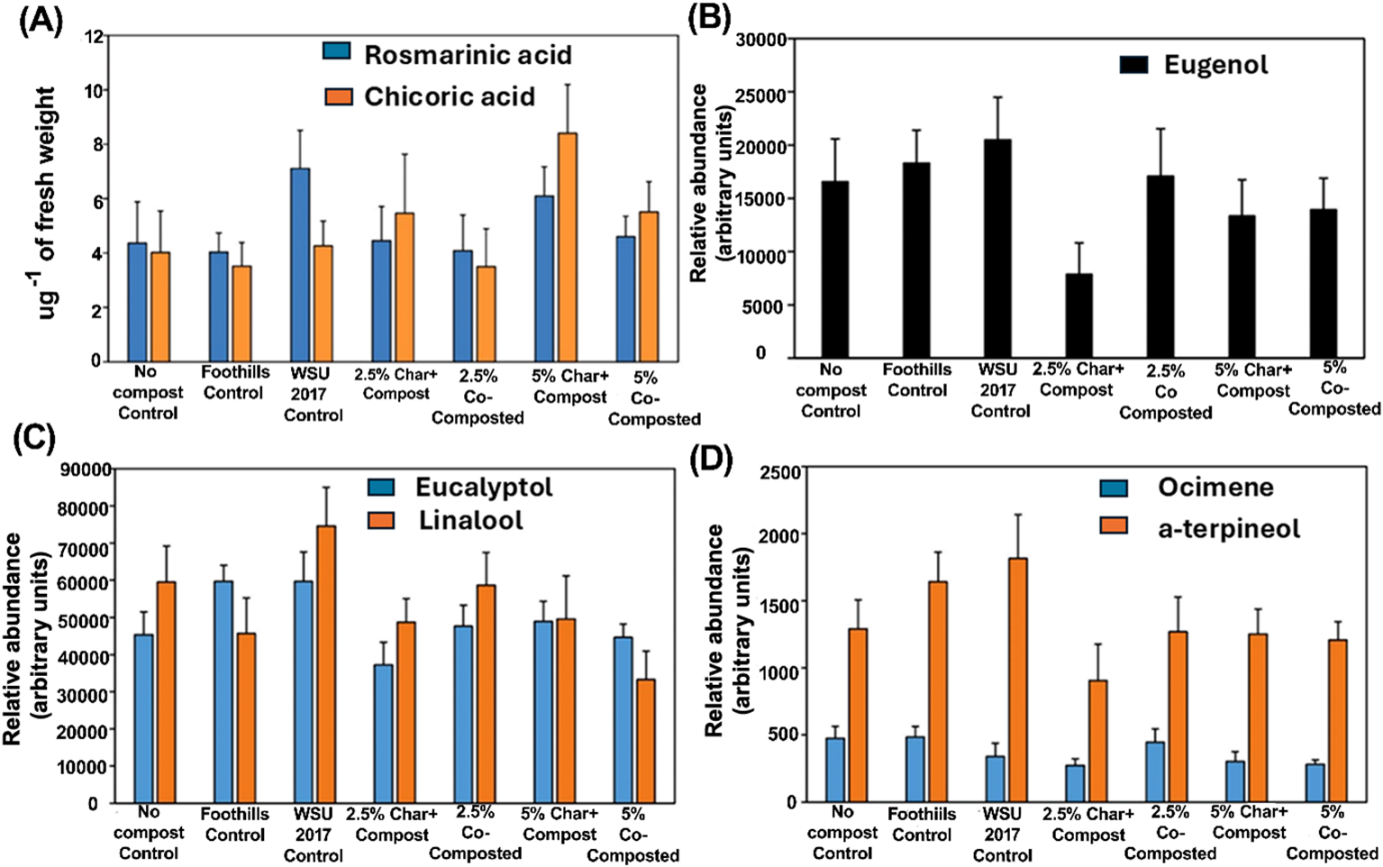
Production of non-volatile antioxidants and volatile aroma compounds in two youngest mature leaf pairs of Genovese sweet basil grown at Footehills Farm (Colbert, Washington). Accumulation of **(A)** major phenolics rosmarinic acid (RA) and chicoric acid (ChA), **(B)** eugenol, **(C)** eucalyptol and linalool, **(D)** ocimene and α-terpineol. Y-axis values are expressed as µg g−1 fresh weight for **(A)**, and as relative abundance (arbitrary units), normalized to internal standard and tissue weight for **(B–D)**. Note that in the treatment labels, “char + compost” indicates char added post-composting, while co-composted means that char was added before composting. Values represent mean ± SEM (n = 10). No statistically significant differences among treatments were detected according to one-way ANOVA (p < 0.05).

The two youngest fully expanded leaf pairs of the harvested plants were further analyzed for their content of major antioxidant phenolics, rosmarinic acid and chicoric acid, and for the levels of aroma-defining volatile compounds. In the Genovese variety used here, those are eugenol, linalool, and eucalyptol. The levels of rosmarinic acid and chicoric acid showed substantial biological variation within and between treatments, as reflected by large error bars (**Figure 5A**). Due to the large error bars, it could not be concluded that these compounds differed in response to the different treatments. Thus, application of various biochar-based treatments did not appear to affect the phenolic compound composition of the basil plants. For volatile flavor components, the same pattern emerged, where no differences in compound abundance were observed in response to the different treatments (**Figures 5B–D**).

When the data were analyzed using one-way ANOVA (p < 0.05), no significant differences were detected. At the same time, pairwise two-tail t-tests, which only compare between two selected treatments, suggested that some differences could be measured. For example, the concentration of linalool decreased in plants grown with 5% co-composted biochar as compared to plants treated with unamended compost (**Figure 5C**). Notably, some plants exhibited a deviation from the major chemotype, accumulating methyl chavicol instead of eugenol. The largest number of plants with the methyl chavicol chemotype (5 of 10) was measured in plants grown with 2.5% non-composted biochar added to WSU 2017 compost. This deviation appeared sporadic and was not consistently linked to any specific treatment.

#### 3.1.3 Strawberry greenhouse trial

**Figure 6:**
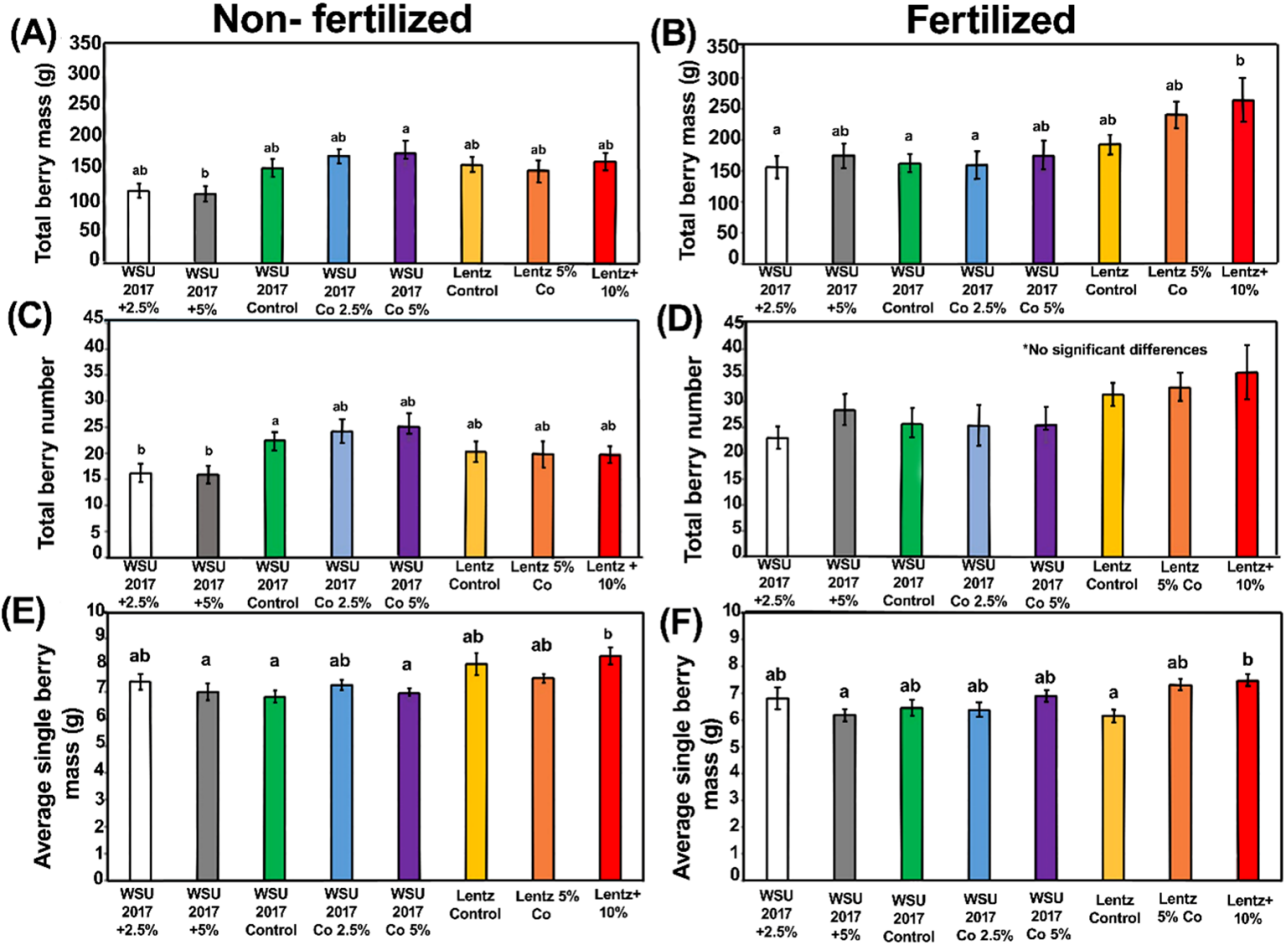
Effect of compost and biochar amendments on the productivity of Albion strawberries. Two cohorts of plants (fertilized and non-fertilized) were subjected to 8 treatments. Fruit was harvested as it ripened over 22 harvests from each plant. **(A, B)** Total berry mass and **(C, D)** berry number per plant were recorded. **(E, F)** Average single berry mass **(E, F)** was calculated from the totals. In these images, “+5%” indicates 5% biochar added after composting, and “5% co” indicates 5% biochar co-composted (added before composting). Results are means ± s.e.m. (n=10). Different letters indicate significant differences between means, calculated by one-way ANOVA with Tukey *post-hoc* tests (p<0.05).

Commercial strawberries (Albion) were grown in pots in the greenhouse. Half of the plants were fertilized, and both fertilized and non-fertilized plants were subjected to eight treatments comparing WSU 2017 and Lenz composts and Oregon Biochar Solutions biochar. The results obtained from the two cohorts (fertilized and non-fertilized) were not identical (**Figure 6**). In the non-fertilized group, the difference was only significant between two treatments (**Figure 6A**). Cumulative berry mass collected from plants grown with WSU 2017 compost that was co-composted with 5% biochar was greater than when the same amount of biochar was added after composting. However, neither of these two treatments differed from the corresponding compost-only yields. None of the treatments with Lenz compost had different effects on cumulative berry mass yield. The total berry number from plants grown with WSU 2017 compost plus biochar (both 2.5% and 5%) was lower in comparison to the compost-only control (**Figure 6C**).

In the fertilized group, supplementation with Lenz compost plus 10% biochar resulted in greater total berry mass as compared to three other treatments (WSU 2017 compost alone or with 2.5% co-composted biochar or biochar added after composting) (**Figure 6B**). The difference was not significant in comparison to other amendments with compost generated at Lenz Enterprises. The cumulative berry number did not vary between treatments (**Figure 6D**). For both fertilized and non-fertilized groups, the average single berry mass from plants grown with Lenz compost plus 10% biochar was greater in comparison to several other treatments (**Figures 6E**, **F**).

#### 3.1.4 Strawberry field trial

**Figure 7:**
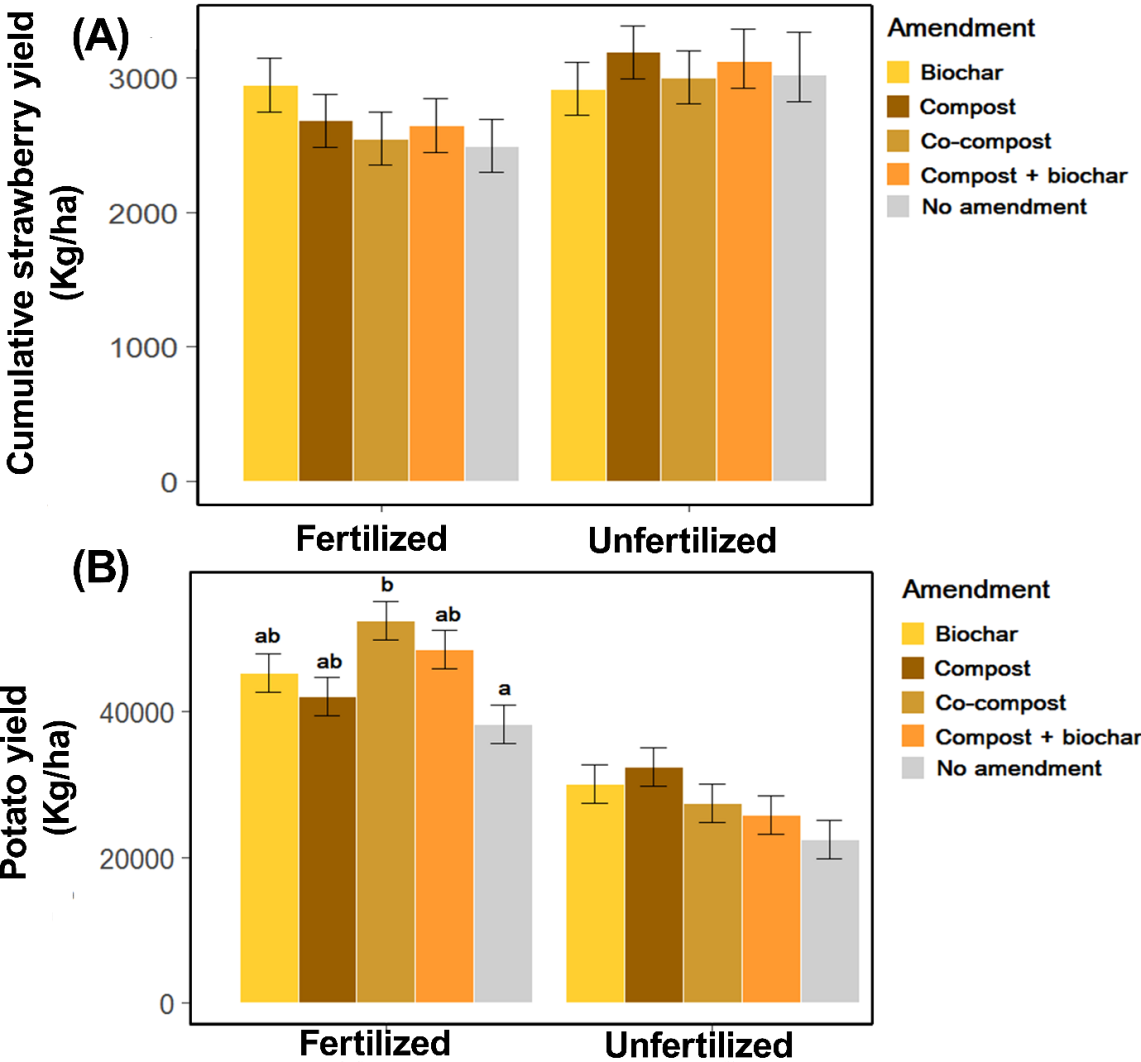
Effects of fertilizer and soil amendments on strawberry and potato yield. **(A)** Cumulative strawberry yield and **(B)** potato yield. Values represent mean ± SEM. Different letters indicate statistically significant differences according to one-way ANOVA followed by Tukey’s HSD test (p < 0.05).

Strawberry yields, measured as cumulative production throughout one season (kg·ha⁻¹), did not increase in response to fertilizer applications, nor were they affected by any of the surface-applied soil amendments (**Figure 7A**). Plants across all treatments produced comparable yields, and no significant differences were detected between fertilized and non-fertilized plots. Similarly, none of the compost or biochar-based treatments altered cumulative fruit mass per hectare. These results indicate that, under the conditions of this field experiment, strawberry yield performance remained uniform regardless of fertilization status or amendment type during the first year of establishment.

#### 3.1.5 Potato field trial

Potato yields, in contrast to strawberry yields, were strongly affected by fertilizer treatment (**Figure 7B**). Average tuber yields in fertilized plots increased by 113% compared to the unfertilized split plots, confirming a clear positive response to supplemental nitrogen. Across all plots, fertilization consistently resulted in larger tuber mass per unit area. Differences within the amendment factor were also observed, but only in fertilized plots. In these cases, tuber yields from co-compost amended plots were higher than those from unamended controls (**Figure 7B**). No significant differences among amendment treatments were detected in the unfertilized plots, where yields remained uniformly low. These results indicate that the positive effect of co-compost amendment on potato productivity was expressed only when adequate nitrogen was supplied, whereas in the absence of fertilizer inputs, amendment effects were negligible.

## 4 Discussion

This study provides new insights into the role of compost, biochar, and biochar-compost or co-composted biochar amendments in specialty crop systems by comparing their impacts on basil, strawberry, and potato under greenhouse and field conditions. Across experiments, co-composted biochar frequently enhanced biomass or yield, particularly in basil and potato, while strawberry responses were more variable and strongly influenced by environmental context and fertilization. In basil, co-composted biochar increased vegetative biomass without altering the accumulation of key antioxidant phenolics or volatile aroma compounds, confirming earlier reports that biochar amendments improve growth but do not compromise phytochemical quality (Pandey et al., 2016; Gang et al., 2018). The relatively stable accumulation of antioxidant phenolics and volatile aroma compounds despite increased biomass suggests that amendment-induced growth enhancement did not occur at the expense of major specialized metabolites associated with basil quality. This may indicate that carbon assimilation and growth were enhanced without substantially disrupting metabolic partitioning toward specialized metabolites (Fallah et al., 2023).

The cultivar-specific responses observed for basil suggest a genetic basis for amendment responsiveness, consistent with prior findings in cereals and legumes (Agegnehu et al., 2016; Schulz et al., 2013). Such genotype-dependent responses may reflect differences in root architecture, nutrient uptake efficiency, rhizosphere interactions, and carbon allocation strategies among cultivars, which together influence the capacity of plants to utilize amendment-mediated improvements in soil conditions. In strawberries, amendment benefits were evident in greenhouse trials where compost amended with 10% biochar increased berry size and total yield under fertilized conditions, but were not observed in first-year field trials, reflecting evidence that organic amendments often require multiple seasons before yield benefits emerge in perennial systems (Latrou and Papadopoulos, 2016; Vestberg et al., 2008). Enhanced fruit yield under some amendment treatments may reflect improved source–sink relationships resulting from greater nutrient availability and more stable root-zone conditions, which can influence carbohydrate allocation and fruit development. Moreover, environmental stressors such as high temperatures at transplanting likely reduced flower initiation and masked potential amendment effects, consistent with earlier work on strawberry physiology (Manakasem and Goodwin, 2001). These observations further suggest that the effectiveness of biochar-based amendments may depend strongly on environmental buffering capacity, where improvements in water retention and nutrient availability are insufficient to overcome severe temperature-induced developmental constraints.

In contrast, potatoes exhibited clear yield improvements with co-composted biochar, but only in fertilized plots, suggesting that biochar functions primarily by improving nutrient-use efficiency rather than directly supplying nutrients, a mechanism supported by previous studies showing that biochar enhances nutrient retention and availability while altering soil chemical cycling (Hagemann et al., 2017; Kammann et al., 2015). This interpretation is supported by the relatively high carbon-to-nitrogen ratio of the biochar, indicating that the amendment itself was unlikely to function as a major nitrogen source during the experimental period. Instead, co-composting likely enhanced nutrient retention and nutrient synchrony within the soil system, thereby improving fertilizer-use efficiency under high nutrient demand conditions. Improved nutrient retention and nutrient synchrony within fertilized soils may have supported greater metabolic demand during tuber development, particularly for carbon and nitrogen assimilation processes associated with storage organ formation.

The observed responses likely arise from coordinated changes in rhizosphere physicochemical conditions rather than from direct nutrient addition alone. Biochar possesses high surface area, porosity, and charge density, which can enhance adsorption and gradual release of mineral nutrients while simultaneously reducing nutrient leaching losses. During co-composting, organic compounds and nutrients can become associated with biochar surfaces, thereby enriching biochar surfaces with nutrients and modifying their surface functionality. This process may improve cation exchange capacity, increase nutrient retention within the root zone, and create microsites that support microbial colonization and metabolic activity. Moreover, co-composted biochar may function not only as a nutrient-retention matrix, but also as a regulator of rhizosphere moisture and aeration. These combined effects may collectively influence microbial interactions and plant productivity (Mikajlo et al., 2024). Many of the observed productivity responses are also likely linked to changes in plant metabolic status resulting from altered rhizosphere conditions. Improved nutrient retention, water availability, and microbial activity can influence carbon allocation, nitrogen assimilation, osmotic regulation, and respiratory energy balance, thereby affecting biomass accumulation and reproductive development. In this context, co-composted biochar may indirectly support more stable metabolic functioning under fluctuating environmental conditions by moderating resource availability within the root zone.

Mechanistically, co-composting biochar with organic matter improves its surface chemistry and nutrient-charging capacity, buffers soil pH, enhances water-holding capacity and aggregate stability, and provides microbial habitat, resulting in improved plant–soil interactions (Agegnehu et al., 2017; Bonanomi et al., 2017; De Tender et al., 2016a, b). The variable responses among these crops highlight the importance of crop-specific physiology and management context where amendment-induced improvements in rhizosphere conditions may differentially influence metabolic allocation and productivity depending on crop type. Leafy annuals such as basil may respond rapidly to nutrient availability and primarily allocate carbon toward vegetative and specialized metabolite production, while perennial fruit crops such as strawberry require longer-term soil conditioning to realize benefits and depend strongly on reproductive source–sink coordination, and nutrient-demanding tuber crops such as potato may benefit most when biochar–compost amendments are combined with fertilization because tuber crops require sustained carbon and nutrient flux toward storage organ development. Collectively, these results suggest that biochar–compost amendments influence crop performance not only through modification of soil physicochemical properties, but also through indirect effects on plant metabolic balance and resource allocation that are governed not by a single factor, but by interactions among amendment chemistry, soil properties, crop physiology, nutrient availability, metabolic balance, and environmental conditions.

These findings have important implications for specialty crop production: co-composted biochar appears to offer the most consistent yield benefits while maintaining crop quality, but responses vary across crops and management systems, underscoring the need for tailored recommendations. The generally stronger performance of co-composted biochar relative to post-compost biochar addition suggests that physicochemical interactions occurring during the composting process itself may be critical for generating agronomically effective amendment properties (Mikajlo et al., 2024). A limitation of this study is that it was conducted over a single growing season, whereas many of the structural and microbial changes induced by biochar accumulate over longer timescales (Agegnehu et al., 2017; Oldfield et al., 2018), suggesting that multi-year trials are required to fully assess amendment impacts. Future research integrating soil physicochemical measurements, microbial community analyses, and crop quality assessments will be crucial to uncover mechanisms and optimize amendment strategies. Overall, our results contribute to a growing body of evidence that compost and biochar amendments, particularly when co-composted, can enhance yield and soil function in specialty crop systems, but that outcomes are strongly dependent on amendment type, crop, and agronomic context (Bird et al., 2012; Schulz et al., 2013; Wang et al., 2017). For specialty crop growers, our findings suggest that co-composted biochar provides the most reliable yield benefits without compromising product quality. However, the effectiveness of these amendments varies by crop type, management system, and environmental conditions. Biochar applied alone may offer limited short-term yield benefits, whereas co-composted formulations consistently enhanced productivity in basil and potato and improved fruit size in greenhouse-grown strawberries. Given that many biochar benefits, particularly those related to soil structure and microbial ecology, accrue over time, multi-year field studies are essential to develop robust recommendations (Agegnehu et al., 2017; Oldfield et al., 2018). Future work should also integrate soil chemical and biological analyses, explore different biochar feedstocks and pyrolysis conditions, and conduct life-cycle and economic assessments to ensure that biochar–compost technologies are agronomically effective, environmentally sustainable, and economically viable.

## 5 Conclusion

Amendment with compost, co-composted biochar, and compost plus biochar resulted in some productivity increase in sweet basil and strawberries. However, the effects were not uniform and varied by compost, biochar, and crop. The same amendments to the soil did not affect the phytochemical composition of field- or greenhouse-grown sweet basil. For the potato field trial, co-compost amendments were the only amendment whose application resulted in crop yield increases, but this was demonstrated only in fertilized split-plots. The use of biochar as a soil amendment in cropping systems may be beneficial, but its use and the intent of its use need to be carefully considered and clearly defined. The differences we observed between biochar products (i.e., co-composted biochar or biochar alone) warrant this consideration, as growers using one or the other will likely see drastic differences in performance, and so, expectations for yield and soil responses should be appropriate to the product. Finally, it is important to note that the data presented here is from one growing season, which makes it difficult to draw conclusions and make confident statements. A second season of data collection would allow us to further evaluate the potential use of biochar and co-composted products as soil amendments, thereby improving recommendations to interested users. Repeated field trials, which are planned for the upcoming years, will determine the impact of weather and other variable conditions on the treatments. It should also be noted that because the characteristics of the biochar and compost impact chemical and physiological processes in the plant, the use of different types of biochar or compost in these studies would be expected to yield different results.

## Data Availability

The original contributions presented in the study are included in the article/supplementary material. Further inquiries can be directed to the corresponding author.
