# Supplementary material for: Biochar and compost amendments promote yield in specialty crops
Source: Front Plant Sci. 2026 Jun 19;17:1849840. doi: 10.3389/fpls.2026.1849840 (PMC13327866; doi:10.3389/fpls.2026.1849840)
Supplement: Supplementary file 1 [file Table1.docx]

**Supplementary tables**

Table S1: Selected chemical properties for biochar (OBS), compost (Lenz), and co-compost.

| **Amendment** | **Carbon**  **(wt. %)** | **Nitrogen**  **(wt. %)** | **Moisture**  **(wt. %)** | **Ash**  **(wt. %)** | **C:N**  **ratio** |
| --- | --- | --- | --- | --- | --- |
|  |  |  |  |  |  |
| Biochar | 87 | 0.78 | 15.0 | 11.2 | 112 |
| Compost | 32 | 2.2 | 52.1 | 38.1 | 15 |
| Co-compost | 32 | 2.2 | 52.7 | 38.9 | 15 |

Table S2: Summary of biochar and compost sources used in the study.

| **Experiment** | **Biochar source** | **Compost source** | **Measurements** |
| --- | --- | --- | --- |
| Basil – field (Colbert) | Amaron Energy | WSU 2017  Footehills | fresh plant mass  phytochemical composition |
| Basil – greenhouse | Amaron Energy  OBS | WSU 2017  WSU 2018  Lenz | fresh plant mass  phytochemical composition |
| Strawberry – greenhouse | Amaron Energy | WSU 2017 | yield  berry number  single berry mass |
| Strawberry – field  (Puyallup) | OBS | Lenz | yield |
| Potato – field  (Mount Vernon) | OBS | Lenz | yield |

Table S3: Summary of treatments used in the basil greenhouse experiment.

| **Treatment #** | **Compost** | **Biochar** | **%** | **Co-composted or biochar+compost** |
| --- | --- | --- | --- | --- |
| 1 | WSU 2017 | None | 0.0 | n/a |
| 2 | WSU 2017 | Amaron Energy | 2.5 | Co-compost |
| 3 | WSU 2017 | Amaron Energy | 5.0 | Co-compost |
| 4 | WSU 2018 | None | 0.0 | n/a |
| 5 | WSU 2018 | OBS | 2.5 | Co-compost |
| 6 | WSU 2018 | OBS | 5.0 | Co-compost |
| 7 | WSU 2018 | OBS | 10.0 | Co-compost |
| 8 | WSU 2018 | OBS | 2.5 | Biochar+compost |
| 9 | WSU 2018 | OBS | 5.0 | Biochar+compost |
| 10 | WSU 2018 | OBS | 10.0 | Biochar+compost |
| 11 | Lenz | None | 0.0 | n/a |
| 12 | Lenz | OBS | 5.0 | Co-compost |
| 13 | Lenz | OBS | 5.0 | Biochar+compost |

Table S4: Product, analysis, and rate for fertilized potato plots in Mount Vernon, Washington.

| **Product and analysis** | **Rate (kg ha^-1^)** |
| --- | --- |
| Ammonium sulfate (20.5-0-0) | 89.67 |
| Monoammonium phosphate (11-52-0) | 67.25 |
| Urea (46-0-0) | 196 |
| Ammonium phosphate (10-34-60) | 67.25 |
